# Application of the information–motivation–behavioral skills model in rehabilitation training for stroke patients

**DOI:** 10.3389/fneur.2026.1709375

**Published:** 2026-03-10

**Authors:** Yingying Peng, Rue Zheng, Wenjie Gan, Limian Feng, Yanzhen Zhai

**Affiliations:** 1 Department of Gynecology, The Fifth Affiliated Hospital, Southern Medical University, Guangzhou, China; 2 Department of Respiratory Medicine, The Fifth Affiliated Hospital, Southern Medical University, Guangzhou, China; 3 Department of Neurology, The Fifth Affiliated Hospital, Southern Medical University, Guangzhou, China; 4 Department of Nursing, The Fifth Affiliated Hospital, Southern Medical University, Guangzhou, China

**Keywords:** caregiver burden, IMB model, multidisciplinary intervention, self-efficacy, stroke rehabilitation

## Abstract

**Objective:**

This study aimed to evaluate a multidisciplinary rehabilitation program based on the information–motivation–behavioral skills (IMB) model principles and its association with self-efficacy, functional recovery, quality of life, and caregiver burden among stroke survivors.

**Methods:**

A quasi-experimental, non-randomized controlled trial was conducted on 112 stroke patients. The IMB group received a 3-month IMB-based program integrating neurologists, rehabilitation therapists, psychologists, and caregivers, and focusing on information delivery, motivational interviewing, and personalized behavioral training. The usual-care group received standard care. The outcomes included self-efficacy (SSEQ), motor function (Fugl–Meyer Assessment, FMA), daily living ability (Barthel Index, BI), quality of life (SS-QOL), psychological status (Hamilton Depression Rating Scale, HAMD; Hamilton Anxiety Rating Scale, HAMA), and caregiver burden (ZBI), assessed at baseline and post-intervention.

**Results:**

The IMB group achieved higher scores than the usual-care group in the following areas: self-efficacy (+82.5% from baseline; SSEQ: 82.5 ± 7.3 vs. 57.8 ± 8.1; *p* < 0.001), motor function (+79.4%; FMA: 68.9 ± 10.2 vs. 50.3 ± 9.5; *p* < 0.001), and quality of life (+71%; SS-QOL: 89.4 ± 11.6 vs. 65.2 ± 10.9; *p* < 0.001). Anxiety (HAMA: 7.5 ± 2.8 vs. 13.6 ± 3.5) and depression (HAMD: 9.2 ± 3.1 vs. 14.8 ± 4.2) scores were lower in the IMB group and fell within the subclinical range (*p* < 0.001), and caregiver burden was also lower in the IMB group (−31%; ZBI: 28.4 ± 6.3 vs. 41.2 ± 7.1; *p* < 0.001).

**Conclusion:**

The IMB-based multidisciplinary intervention was associated with improved stroke recovery outcomes and reduced caregiver stress. This model suggests a potentially scalable approach that warrants further investigation. Its integration of behavioral strategies with neurorehabilitation principles bridges a critical gap in holistic stroke care, emphasizing the importance of self-efficacy and multidisciplinary collaboration.

## 1 Introduction

Stroke, a leading cause of mortality and long-term disability worldwide, places a significant burden on healthcare systems and the affected families (1). In China, approximately 3.4 million new stroke cases are reported annually, with more than 70% of survivors experiencing persistent motor dysfunction and reduced quality of life (2, 3). Despite advancements in acute stroke management, post-stroke rehabilitation remains suboptimal, particularly in low-resource settings, in which patient adherence to rehabilitation protocols and caregiver support are critical challenges (4, 5).

Recent studies have emphasized the role of behavioral interventions in addressing these challenges (6, 7). The information–motivation–behavioral skills (IMB) model, initially developed for HIV prevention, has shown promise in chronic disease management by addressing knowledge gaps, enhancing motivation, and fostering actionable behavioral changes (8). Although the IMB model has demonstrated effectiveness in improving outcomes in the management of diabetes and hypertension, its application to stroke rehabilitation is novel. Unlike medication adherence in chronic diseases, stroke recovery requires motor skill relearning, caregiver involvement, and multidisciplinary coordination. Our study uniquely integrates IMB principles with neuroplasticity-based training and caregiver support, addressing these stroke-specific challenges that were not present in prior chronic disease applications (7, 9). Recent studies have recommended its applicability to stroke rehabilitation, where patient self-efficacy and sustained engagement in physiotherapy are pivotal for functional recovery (10). For instance, interventions integrating IMB principles have improved exercise adherence and medication adherence in stroke survivors and reduced their National Institutes of Health Stroke Scale (NIHSS) scores (11). However, existing research predominantly focuses on isolated outcomes (e.g., motor function) and lacks a multidisciplinary approach, limiting its translational impact.

Compounding these issues, stroke recurrence and comorbidities—such as hypertension and diabetes—remain poorly managed in clinical practice (12, 13). A 2025 study demonstrated that dietary modifications, including low-sodium salt substitution, reduced the risk of stroke recurrence by 14% and mortality by 12%, highlighting the need for holistic, patient-centered interventions (14). Concurrently, emerging evidence has highlighted the roles of neuroplasticity and vascular regeneration in post-stroke recovery; however, few studies bridge these biological mechanisms with behavioral interventions (15).

This study aims to address these gaps by evaluating a multidisciplinary IMB-based rehabilitation program for stroke patients. By integrating neurologists, rehabilitation therapists, psychologists, and caregivers, we hypothesize that this approach will be associated with increased self-efficacy, improved motor function, and enhanced quality of life, while alleviating psychological distress and caregiver burden. Our study builds on prior findings that early, structured rehabilitation improves cortical reorganization and functional outcomes (16) but innovates by embedding IMB principles into a coordinated care framework tailored to resource-limited settings.

## 2 Methods

### 2.1 Study design and participants

This quasi-experimental study used a non-randomized controlled design with baseline and follow-up assessments to examine the associations between an IMB model-aligned rehabilitation program and outcomes in stroke patients. A total of 112 participants were recruited from the Department of Neurology at the Fifth Affiliated Hospital of Southern Medical University between April 2024 and November 2024. To minimize contamination between groups, non-concurrent allocation was utilized: patients admitted from April to July 2024 were assigned to the usual-care group (*n* = 56), while those admitted from August to November 2024 formed the IMB group (*n* = 56).

The inclusion criteria required participants to be aged 18–75 years, diagnosed with a first-time stroke (ischemic or hemorrhagic) within 1 month, exhibiting stable vital signs and limb motor dysfunction (Fugl–Meyer motor sub-score ≤ 84/100), and without severe aphasia (Boston Diagnostic Aphasia Examination Severity Rating Scale ≤ 2, i.e., “moderate” or better; able to produce ≥ 50% intelligible responses on a 10-item picture-naming task). The exclusion criteria included recurrent stroke, pre-existing central or peripheral motor disorders (e.g., Parkinson’s disease, cerebral palsy, and peripheral neuropathy), orthopedic conditions that independently limit active or passive range of motion or weight-bearing (e.g., fractures, joint arthrodesis, and severe contractures), psychiatric disorders, or terminal illnesses.

To minimize internal validity threats, we implemented comprehensive bias controls, including standardized protocols with identical inclusion criteria across both periods, assessor blinding with trained staff isolated from the IMB group, temporal monitoring that confirmed no hospital policy changes between April–July (usual-care) and August–November (IMB) periods, and consistent data collection using identical questionnaires with uniform timing (±7 days). The IMB intervention followed a written manual with monthly fidelity checks yielding 94.2% adherence.

Sample size calculation, based on a 7-point improvement in quality of life (SS-QOL) with 90% power and *α* = 0.05, determined 40 participants per group, which was adjusted to 56 per group after accounting for a 20% attrition rate.

The study protocol was approved by the Ethics Committee of the Fifth Affiliated Hospital of Southern Medical University (Approval No.: 2024-HLB-K-003). Written informed consent was obtained from all participants and their caregivers prior to enrollment.

### 2.2 Intervention protocol

The usual-care group received standard neurological care, encompassing (1) bedside rehabilitation training initiated 48-h post-stabilization, including physical therapy (passive/active limb movements, 2 sessions/week × 45 min, 18 h total) and occupational therapy (ADL training, 12 sessions × 30 min, 6 h total); (2) medication adherence education (single 30-min session); (3) discharge instructions; (4) three follow-up telephone calls (at weeks 1, 2, and 4 post-discharge, totaling approximately 1.5 h); and (5) baseline and 3-month assessments (2 h). Total contact time was approximately 28 h over 12 weeks. To maintain ethical standards while controlling for attention, the usual-care group received three non-interactive educational videos (45 min total) after completing outcome assessments.

The IMB group received usual care plus structured IMB-specific components. The information support involved tailored individual counseling (5–6 sessions × 30 min during hospitalization, approximately 2.5–3 h), weekly group health lectures via video conference (12 sessions × 60 min, 12 h), and educational videos (12 videos × 15 min, 3 h) provided during the intervention period. Motivational interviewing was delivered by Motivational Interviewing Network of Trainers (MINT) nurses using a 5-stage protocol (pre-contemplation, contemplation, preparation, action, and maintenance) over 12 weeks (approximately 3 h). Family involvement was standardized through four structured sessions (weeks 1, 3, 6, and 10; 4 sessions × 45 min, 3 h total), covering recovery education, emotional support techniques, and hands-on exercise assistance (Supplementary Table 1). Behavioral skills training featured personalized rehabilitation plans designed by a multidisciplinary team, including rehabilitation therapists, psychologists, and nutritionists. The intervention included enhanced physical therapy (3 sessions/week × 60 min, 36 h total); occupational therapy (2 sessions/week × 45 min, 18 h total); psychological support using cognitive-behavioral techniques (12 sessions × 60 min, 12 h); nutritional counseling by registered dietitians (2 sessions × 30 min, 1 h); and hands-on caregiver skills training in safe transfer techniques, medication management, and home exercise assistance, integrated into family sessions. Post-discharge follow-up was reinforced through weekly one-to-one remote supervision via the WeChat platform (approximately 6 h over 12 weeks). The total contact time for the IMB group was approximately 96.5 h over 12 weeks, comprising approximately 28 h of usual care plus approximately 68.5 h of incremental IMB-specific components beyond usual care (Supplementary Table 2).

### 2.3 Outcome measures

Outcomes were assessed at baseline and 3-month post-intervention using the following validated scales: self-efficacy (Stroke Self-Efficacy Questionnaire, SSEQ) (17), motor function (Fugl–Meyer Assessment, FMA) (18), quality of life (Stroke-Specific Quality of Life Scale, SS-QOL) (19), daily living ability (Barthel Index, BI: 10 items—feeding, bathing, grooming, dressing, bowels, bladder, toilet use, transfers, mobility, stairs—each scored 0/5/10, total 0–100 (with 100 indicating full independence); inter-rater reliability *κ* ≥ 0.91) (20), psychological status [Hamilton Depression (HAMD) and Anxiety (HAMA) Rating Scales] (21), and caregiver burden (Zarit Burden Interview, ZBI) (22). Although multiple endpoints were assessed, the SS-QOL was pre-specified as the primary outcome for sample-size calculation and is hereafter referred to as the primary measure.

All outcome measures were administered by trained assessors who were blinded to group allocation. Scale psychometric properties and minimal clinically important differences (MCIDs) are detailed below:

SSEQ: We administered the 13-item validated scale measuring confidence in post-stroke daily activities. Score range: 13–130; Cronbach’s *α* = 0.92; test–retest ICC = 0.88; MCIDs = 10 points.

FMA: We administered the full 33-item Fugl–Meyer Assessment (23), yielding a 0–100 total score that combines the 66-point upper-limb and 34-point lower-limb subscales while retaining the four deep-tendon-reflex items because they are integral to the original metric and our assessors were trained to elicit them safely. The scale’s psychometric properties include a test–retest ICC of 0.94, an inter-rater ICC of 0.96, a Cronbach’s *α* of 0.95, and a MCID of 5 points (24, 25). To ensure repeatability, two licensed physiotherapists who remained blinded to group allocation completed a 4-h training module that included a video atlas and practice on 10 pilot patients until they achieved a *κ* ≥ 0.90 against a gold-standard neurologist, followed by monthly recalibration sessions that maintained a κ ≥ 0.88 throughout the trial.

SS-QOL: We administered the 49-item, validated stroke-specific quality of life measure. Score range: 49–245; Cronbach’s *α* = 0.89; test–retest ICC = 0.91; MCIDs = 7 points. The MCIDs for the SS-QOL total score were set at 7 points, based on anchor-based validation by Fugl-Meyer et al. (23), who determined that a ≥ 7-point change best discriminated “minimal improvement” from “no change” in 236 stroke survivors (sensitivity: 0.81 and specificity: 0.76). This threshold was used for sample-size calculation and interpretation of clinical relevance in the present study.

BI: We administered the 10-item activities of daily living scale. Score range: 0–100; inter-rater reliability *κ* = 0.91; MCIDs = 10 points.

HAMD-17: We administered the 17-item depression assessment; internal consistency *α* = 0.88; clinical threshold ≥17.

HAMA: We administered the 14-item anxiety assessment; internal consistency α = 0.90; clinical threshold ≥14.

ZBI: We administered the 22-item caregiver burden scale. Score range: 0–88; Cronbach’s α = 0.87.

All scales were administered via a face-to-face interview at baseline and the 3-month follow-up. Assessment fidelity was maintained through standardized training and inter-rater reliability checks (*κ* ≥ 0.85 for all raters).

### 2.4 Data collection and analysis

Trained researchers collected data through face-to-face interviews and electronic questionnaires, with quality control implemented via EpiData 3.1 forced-response fields, range checks, and double data entry; any inconsistencies were resolved within 48 h during weekly calibration meetings. Statistical analysis was performed in R 4.3.0. After screening continuous baseline variables for normality (Shapiro–Wilk and Q–Q plots), non-normal variables (age, NIHSS, baseline SS-QOL, and baseline ZBI) were either log-transformed or analyzed with Mann–Whitney U or Wilcoxon signed-rank tests. Potential demographic and *a priori* covariates—age, sex, stroke type, baseline NIHSS, baseline SS-QOL, baseline BI, baseline FMA, baseline SSEQ, baseline HAMD/HAMA, baseline ZBI, and time-from-onset—were evaluated in univariate screening, and those with a *p* < 0.10 (age, baseline SS-QOL, baseline FMA) were retained in the final ANCOVA models for each outcome. Attrition was 2.7% (IMB 2, usual-care 1); Little’s MCAR test yielded *p* = 0.42, and the missing values were imputed with fully conditional specification multiple-imputation (*m* = 20 iterations) using the mice package, with complete-case sensitivity analyses reported. Between-group changes were assessed with independent *t*-tests (or Mann–Whitney tests) for continuous outcomes and χ^2^ or Fisher’s exact tests for categorical variables, while within-group changes were evaluated with paired *t*-tests (or Wilcoxon tests); effect sizes are presented as mean differences with 95% CIs, and a two-tailed *p* < 0.05 was considered significant.

## 3 Results

### 3.1 Baseline data for enrolled patients

The full study sample comprised 112 stroke patients with complete baseline data (IMB group *n* = 56, standard usual-care group *n* = 56) who were enrolled consecutively from April to November 2024. Baseline characteristics for the complete sample showed comparable profiles between groups with no statistically significant differences in any demographic or clinical variable (all *p* > 0.05). Unless otherwise stated, all between-group comparisons are presented with SS-QOL (the primary outcome) first, followed by the secondary endpoints.

The cohort was predominantly male (IMB group 58.9% vs. usual care group 53.6%, *p* = 0.543) with similar mean ages (62.3 ± 8.5 years vs. 63.1 ± 7.9 years, *p* = 0.612). Stroke classification was balanced with ischemic stroke comprising 76.8% of the IMB group and 73.2% of the standard usual-care group (*p* = 0.832). Disease duration was comparable in 64.3% of the IMB group vs. 60.7% of the usual-care group presenting within 7 days of onset (*p* = 0.456). Stroke severity measured by NIHSS showed no between-group difference (*p* = 0.721), with moderate severity (scores 6–14) observed in 62.5% of the IMB group and 64.3% of the usual-care group. Treatment modalities were uniformly administered, including antiplatelet therapy (91.1% vs. 89.3%) and antihypertensive therapy (85.7% vs. 82.1%, *p* = 0.689). Complication rates were balanced (pneumonia 12.5% vs. 10.7%, urinary tract infection 8.9% vs. 7.1%, *p* = 0.905). Baseline outcome scores showed no significant differences (SSEQ 45.2 ± 6.7 vs. 46.1 ± 7.2, *p* = 0.493; FMA 38.4 ± 9.1 vs. 37.8 ± 8.6, *p* = 0.721; SS-QOL 52.3 ± 10.4 vs. 54.0 ± 9.8, *p* = 0.385) (Table 1).

**Table 1:** Baseline characteristics of participants.

Characteristic	IMB group (*n* = 56)	Usual-care group (*n* = 56)	*P*-value
Demographics			
Age (years), mean ± SD	62.3 ± 8.5	63.1 ± 7.9	0.612
Male patients, n (%)	33 (58.9%)	30 (53.6%)	0.543
Stroke classification			0.832
- Ischemic stroke, n (%)	43 (76.8%)	41 (73.2%)	
- Hemorrhagic stroke, n (%)	13 (23.2%)	15 (26.8%)	
Disease duration			0.456
- ≤ 7 days, n (%)	36 (64.3%)	34 (60.7%)	
- 8–14 days, n (%)	14 (25.0%)	16 (28.6%)	
- 15–30 days, n (%)	6 (10.7%)	6 (10.7%)	
Stroke severity (NIHSS score)			0.721
- Mild (≤5), n (%)	12 (21.4%)	11 (19.6%)	
- Moderate (6–14), n (%)	35 (62.5%)	36 (64.3%)	
- Severe (≥15), n (%)	9 (16.1%)	9 (16.1%)	
Treatment modalities			0.689
- Intravenous thrombolysis, n (%)	18 (32.1%)	16 (28.6%)	
- Endovascular thrombectomy, n (%)	8 (14.3%)	7 (12.5%)	
- Antiplatelet/anticoagulant therapy, n (%)	51 (91.1%)	50 (89.3%)	
- Antihypertensive therapy, n (%)	48 (85.7%)	46 (82.1%)	
Complications			0.905
- Pneumonia, n (%)	7 (12.5%)	6 (10.7%)	
- Urinary tract infection, n (%)	5 (8.9%)	4 (7.1%)	
- Deep vein thrombosis, n (%)	3 (5.4%)	2 (3.6%)	
- Pressure ulcer, n (%)	2 (3.6%)	3 (5.4%)	
Baseline scores			
SSEQ score, mean ± SD	45.2 ± 6.7	46.1 ± 7.2	0.493
FMA score, mean ± SD	38.4 ± 9.1	37.8 ± 8.6	0.721
SS-QOL score, mean ± SD	52.3 ± 10.4	54.0 ± 9.8	0.385

NIHSS, National Institutes of Health Stroke Scale (scores: mild ≤5, moderate 6–14, severe ≥15). SSEQ, Stroke Self-Efficacy Questionnaire; FMA, Fugl–Meyer Assessment; SS-QOL, Stroke-Specific Quality of Life Scale.

### 3.2 Primary outcome

**Figure 1:**
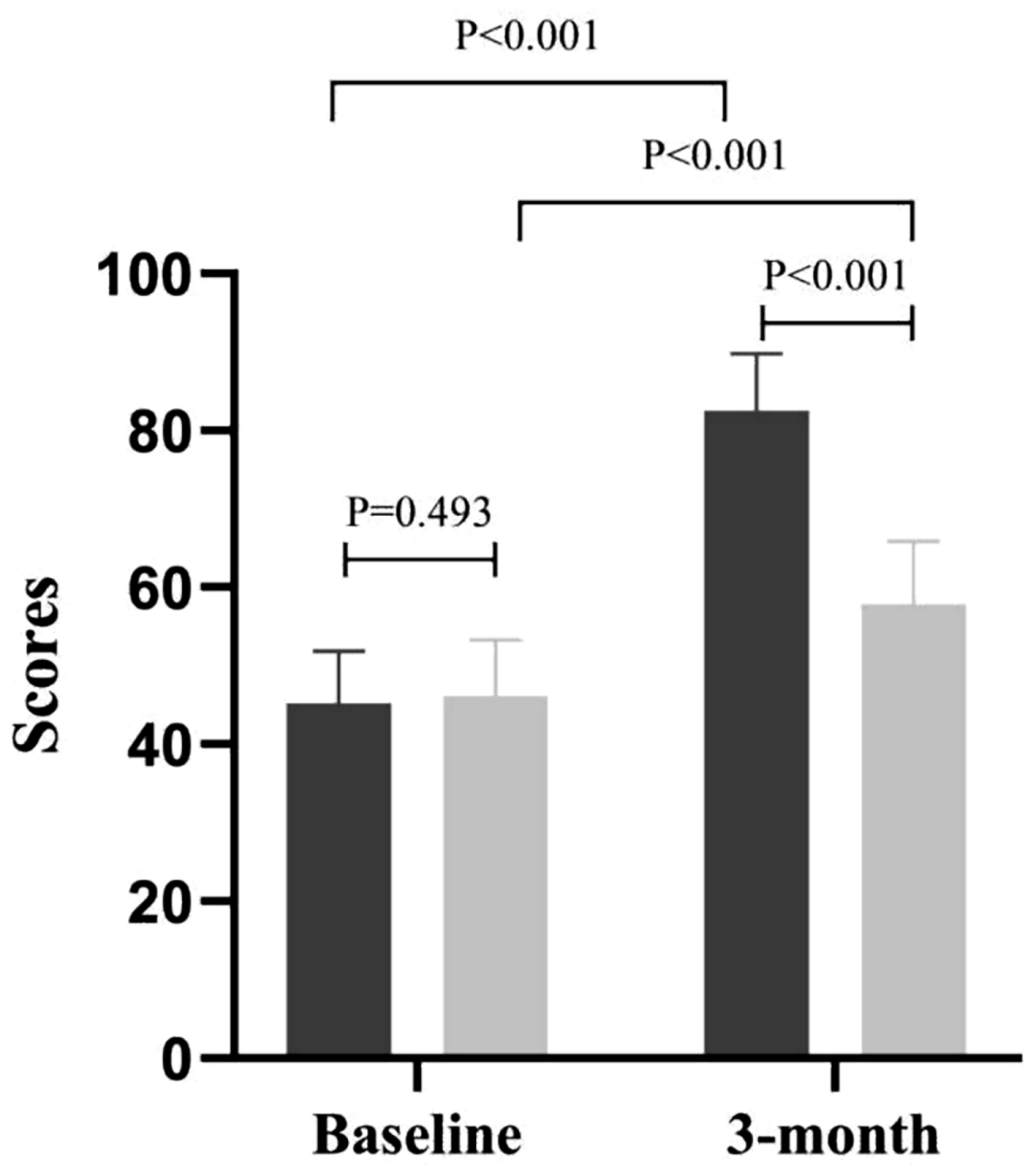
Evaluation of patients’ self-efficacy.

The primary endpoint, SS-QOL, showed a 24.2-point between-group difference (IMB group 89.4 ± 11.6 vs. usual-care group 65.2 ± 10.9; *p* < 0.001), exceeding the 7-point MCIDs. Self-efficacy analysis included 109 participants with complete follow-up data (IMB group *n* = 54 after two subjects were lost to follow-up, standard usual-care group *n* = 55 after one subject was lost to follow-up). At baseline, both groups demonstrated comparable SSEQ scores, as reported in the full sample analysis. Following the 3-month intervention, the IMB group achieved a mean SSEQ score of 82.5 ± 7.3, representing an 82.5% increase from their baseline and exceeding the predefined target threshold. In contrast, the standard usual-care group showed minimal improvement with a mean score of 57.8 ± 8.1. The between-group difference was 24.7 points (95% CI: 21.3–28.1; *p* < 0.001), indicating a significant association between IMB group exposure and self-efficacy improvement (Figure 1). Secondary outcomes are presented in the following subsections.

### 3.3 Assessment of motor function, quality of life, and daily living ability

The motor function assessment included the same 109 participants (IMB *n* = 54, usual-care *n* = 55) with complete FMA data at follow-up. The IMB group achieved a mean FMA score of 68.9 ± 10.2, reflecting a 79.4% improvement from their baseline performance. The usual-care group had smaller changes, with a mean score of 50.3 ± 9.5. The between-group difference reached 18.6 points (95% CI: 14.8–22.4; *p* < 0.001), corresponding to a large effect size (Cohen’s *d* = 2.1) and indicating a significant association between IMB participation and motor recovery.

Quality of life scores were also higher in the IMB group, with a mean SS-QOL score of 89.4 ± 11.6 (a 71% change from baseline) compared to the usual-care group’s 65.2 ± 10.9 (a modest increase from baseline of 54.0 ± 9.8). The between-group advantage for the IMB group was 24.2 points (95% CI: 19.7–28.7; *p* < 0.001), substantially exceeding the SS-QOL MCIDs of 7 points and indicating a strong association between the IMB intervention and quality of life.

**Figure 2:**
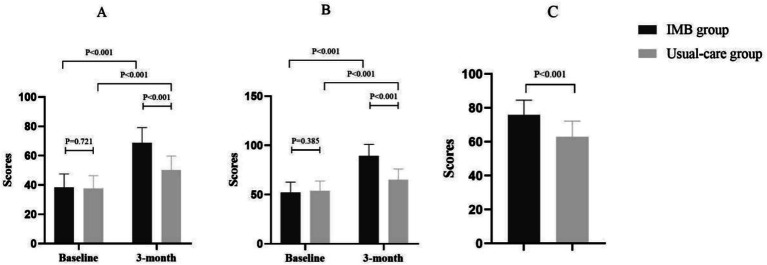
Assessment of motor function **(A)**, quality of life **(B)**, and daily living ability **(C)**.

Daily living ability, as measured by the BI, revealed that the IMB group (*n* = 54) achieved a mean score of 75.8 ± 8.7, transitioning from “needs assistance” to “partial independence,” while the usual-care group (*n* = 55) remained at 62.4 ± 9.3, indicating continued assistance requirements. The between-group improvement difference was 13.4 points (95% CI: 9.8–17.0; *p* < 0.001) (Figure 2).

### 3.4 Evaluation of the patient’s psychological outcomes

**Figure 3:**
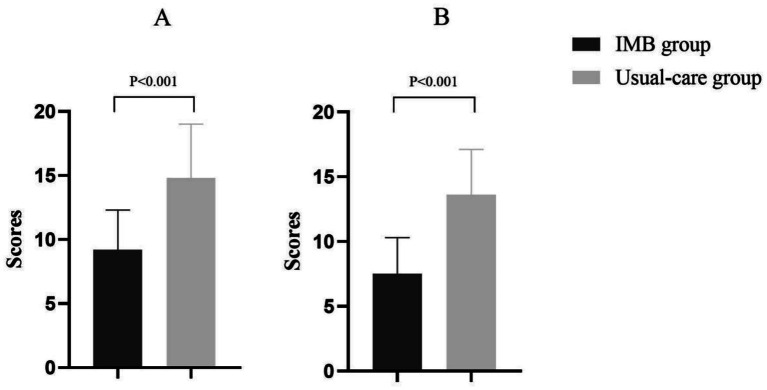
Evaluation of patient post-intervention depression scores (HAMD) **(A)** and anxiety scores (HAMA) **(B)**.

The IMB group showed clinically meaningful reductions in psychological distress. Post-intervention depression scores (HAMD) in the IMB group (9.2 ± 3.1) fell below the clinical threshold (≥17), in contrast to the usual-care group (14.8 ± 4.2; *p* < 0.001). Similarly, anxiety scores (HAMA) decreased to 7.5 ± 2.8 in the IMB group vs. 13.6 ± 3.5 in the usual-care group (*p* < 0.001), indicating subclinical anxiety levels post-intervention (Figure 3).

### 3.5 Comparison of caregiver burden

**Figure 4:**
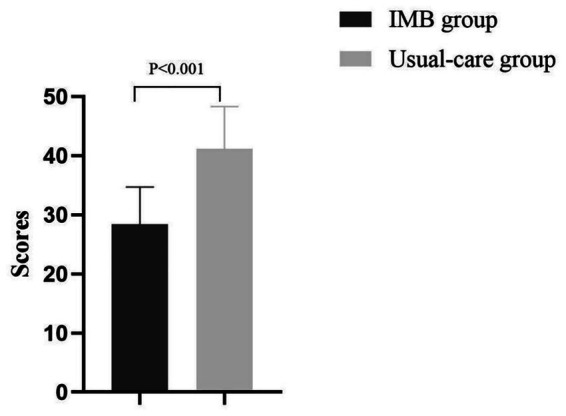
Comparison of caregiver burden.

Caregiver burden, assessed via the ZBI, decreased significantly in the IMB group (28.4 ± 6.3) compared to the usual-care group (41.2 ± 7.1). The IMB group yielded a 31% reduction in caregiver stress, with a mean difference of −12.8 (95% CI: −15.3–-10.3; *p* < 0.001) (Figure 4).

## 4 Discussion

This study found that a multidisciplinary rehabilitation program integrated with the IMB model was associated with higher self-efficacy, functional recovery, and quality of life in stroke survivors, along with lower psychological distress and caregiver burden. The IMB group had an 82.5% increase in self-efficacy (SSEQ) relative to the usual-care group and a 79.4% difference in motor function (FMA). The 24.2-point between-group difference in the primary outcome (SS-QOL) exceeded the 7-point MCID. Differences were also observed for secondary measures (SSEQ, FMA, BI, HAMD/HAMA, and ZBI). These findings are consistent with an association between the integration of behavioral strategies with neurorehabilitation and post-stroke recovery outcomes.

The observed group differences may be consistent with Bandura’s self-efficacy theory (26). “Motivation” via MI likely upregulated dopaminergic signaling, sustaining practice effort despite physical challenges. “Behavioral Skills” provided enactive mastery experiences—repeated successful task completion that drives self-efficacy and directly stimulates activity-dependent neuroplasticity (15). This integrated approach maintained high-intensity practice, crossing the threshold for structural neuroplastic changes. Reduced caregiver burden may further support plasticity by lowering patient stress, which impairs synaptic function.

Structured behavioral training, including personalized goal-setting and motivational interviewing, is likely linked to patients’ confidence in managing rehabilitation tasks, consistent with Bandura’s theory of self-efficacy (26). Similar outcomes were observed in chronic disease management, where IMB-based interventions improved medication adherence and health behaviors (9). Notably, the 31% reduction in caregiver burden (ZBI) underscores the critical role of caregiver education and support, a component often overlooked in resource-limited settings (27). This finding aligns with studies advocating for family-inclusive models to mitigate long-term caregiver strain (28). The IMB group’s success in motor recovery (FMA: 68.9 vs. 50.3) may be linked to task-specific training and neuroplasticity. Repetitive, goal-directed exercises are known to activate cortical reorganization pathways, as demonstrated in models of stroke recovery (29). Our multidisciplinary approach, incorporating mirror therapy and memory drills, likely amplified these biological processes, offering a mechanistic association between behavioral interventions and neurobiological recovery (30). Furthermore, the significant improvement in quality of life (SS-QOL: 89.4 vs. 65.2) reflects the holistic impact of addressing both physical and psychological wellbeing, a priority highlighted in recent stroke care guidelines (31).

The observed differences in motor recovery are consistent with the interplay between behavioral strategies and neurobiological mechanisms. The repetitive, goal-directed exercises in our program likely activated cortical reorganization pathways, such as REF1-mediated vascular regeneration, which is associated with synaptic plasticity (32). Additionally, the IMB model’s focus on information delivery and motivational support may have upregulated dopaminergic signaling in the prefrontal cortex, fostering sustained engagement in rehabilitation tasks (26). This finding aligns with studies showing that interventions combining neurorehabilitation with behavioral nudges improved motor recovery compared to isolated therapies (33). The marked reduction in psychological distress (HAMD: 9.2 vs. 14.8) may be related to the program’s emphasis on social persuasion and mastery experiences, key tenets of Bandura’s self-efficacy theory (26). Similar mechanisms were observed in VR-based interventions, where immersive training reduced anxiety by diverting attention from pain and fostering a sense of control (34).

This aligns with global health initiatives advocating for multidisciplinary stroke care, particularly in regions with high stroke burdens such as China. The program’s reliance on existing healthcare personnel suggests that it may be potentially scalable, although formal implementation studies are needed to assess its feasibility and resource requirements. Future iterations could incorporate dietary interventions, such as low-sodium substitutions, to further reduce recurrence risks—a strategy validated in a 2025 randomized trial (14).

While appropriate for this pilot implementation, our non-randomized design precludes causal inference and introduces potential selection bias despite baseline comparability. The non-concurrent usual-care group, although reducing contamination, may be susceptible to temporal trends and unmeasured confounders (e.g., evolving clinical protocols and seasonal effects). Although we maintained consistent treatment standards and blinded outcome assessment, residual confounding remains possible. Future research should prioritize a multicenter randomized controlled trial to validate these findings, with longer-term follow-up (12 months) to assess the sustainability of benefits and stroke recurrence rates. Our non-concurrent quasi-experimental design presents fundamental limitations, as temporal variations, staff turnover, or seasonal case differences between the April–July and August–November could influence outcomes. The differential data collection burden—IMB weekly WeChat supervision vs. control three brief calls—may independently affect results through attention bias. Non-randomized allocation may have introduced selection bias despite baseline comparability, and we cannot exclude residual confounding by socioeconomic status or digital literacy. The difference in contact time represents an unavoidable confounder that prevents us from disentangling IMB components from simple dose effects. These limitations underscore that our findings demonstrate associations requiring further randomized testing rather than definitive IMB superiority.

## 5 Conclusion

The IMB-based multidisciplinary rehabilitation program was associated with improved stroke recovery outcomes and reduced caregiver burden, suggesting a potentially scalable model. By fostering self-efficacy, leveraging neuroplasticity mechanisms, and engaging caregivers, this approach warrants further investigation to determine its potential for reducing stroke burden. Future studies should focus on longitudinal validation and mechanistic exploration to optimize its translational impact.

## Data Availability

The original contributions presented in the study are included in the article/Supplementary material, further inquiries can be directed to the corresponding author.
