# Correction: Application of the information–motivation–behavioral skills model in rehabilitation training for stroke patients

**DOI:** 10.3389/fneur.2026.1828048

**Published:** 2026-03-25

**Authors:** Yingying Peng, Rue Zheng, Wenjie Gan, Limian Feng, Yanzhen Zhai

**Affiliations:** 1 Department of Gynecology, The Fifth Affiliated Hospital, Southern Medical University, Guangzhou, China; 2 Department of Respiratory Medicine, The Fifth Affiliated Hospital, Southern Medical University, Guangzhou, China; 3 Department of Neurology, The Fifth Affiliated Hospital, Southern Medical University, Guangzhou, China; 4 Department of Nursing, The Fifth Affiliated Hospital, Southern Medical University, Guangzhou, China

**Keywords:** caregiver burden, IMB model, multidisciplinary intervention, self-efficacy, stroke rehabilitation

In the published article, “Dean Fund Project of the Fifth Affiliated Hospital of Southern Medical University, YZ2023ZX10” was erroneously omitted from the **Funding** statement. The updated **Funding** statement appears below.

“**Funding**

The author(s) declared that financial support was received for this work and/or its publication. This work was supported by the Dean Fund Project of the Fifth Affiliated Hospital of Southern Medical University, YZ2023ZX10.”

The original version of this article has been updated.

