# Supplementary material for: Application of the information–motivation–behavioral skills model in rehabilitation training for stroke patients
Source: Front Neurol. 2026 Mar 10;17:1709375. doi: 10.3389/fneur.2026.1709375 (PMC13008678; doi:10.3389/fneur.2026.1709375)
Supplement: Supplementary file 1 [file Table_1.docx]

****Supplementary Table 1. Motivational Interviewing Protocol and Family Engagement Schedule****

| Stage | Week(s) | Patient Session Content | Family Session  (Week) | Core Objectives |
| --- | --- | --- | --- | --- |
| Pre-contemplation | 1 | Awareness building, functional feedback | 1 (45 min) | Establish recovery awareness |
| Contemplation | 2 | Decisional balance, ambivalence resolution | - | Resolve participation ambivalence |
| Preparation | 3 | Goal-setting, action planning | 3 (45 min) | Create collaborative care plan |
| Action | 4-10 | Weekly check-ins, barrier troubleshooting | 6 (45 min) | Reinforce home exercise support |
| Maintenance | 11-12 | Relapse prevention, self-management | 10 (45 min) | Ensure sustainable caregiver strategies |

****Supplementary Table 2.** Detailed Comparison of Intervention Components and Contact Time Between IMB Group and** usual-care Group

| Component | IMB Group  (n=56) | usual-care Group  (n=56) | Notes |
| --- | --- | --- | --- |
| **Information Delivery** |  |  |  |
| Individual counseling | 5-6 sessions × 30 min (2.5-3 h) | 0 sessions | Inpatient phase only |
| Group health lectures | 12 sessions × 60 min (12 h) | 0 sessions | Video conference post-discharge |
| Educational videos | 12 videos × 15 min (3 h) | 3 videos × 15 min (45 min) | Content matched |
| **Motivational Interviewing** |  |  |  |
| Individual MI stages | 5 stages over 12 weeks (3 h) | 0 stages | MINT-certified nurses |
| Family sessions | 4 sessions × 45 min (3 h) | 0 sessions | Standardized manual |
| **Multidisciplinary Therapy** |  |  |  |
| Physical therapy | 36 sessions × 60 min (36 h) | 24 sessions × 45 min (18 h) | 2.4× more contact time |
| Occupational therapy | 24 sessions × 45 min (18 h) | 12 sessions × 30 min (6 h) | 3× more contact time |
| Psychological support | 12 sessions × 60 min (12 h) | 0 sessions | Clinical psychologist |
| Nutritional counseling | 2 sessions × 30 min (1 h) | 0 sessions | Registered dietitian |
| **Follow-up & Supervision** |  |  |  |
| Remote supervision | Weekly WeChat × 12 wks (~6 h) | 3 phone calls (1.5 h) | Different intensity |
| Assessment time | Baseline + 3-month (2 h) | Baseline + 3-month (2 h) | Equivalent |
|  |  |  |  |
| **Total contact time** | **~96.5 hours** | **~28 hours** | **3.4× difference** |

Total contact time includes standard care (approximately 28 hours for both groups, comprising bedside rehabilitation including physical and occupational therapy, medication education, discharge instructions, follow-up calls, and assessments) plus incremental IMB components specific to the IMB group (approximately 68.5 additional hours). To enable direct comparison of intervention intensity, assessment time (2 hours for baseline and 3-month evaluations) is included in the approximately 28 hours usual-care baseline for both groups. The IMB group's approximately 96.5 hours total includes these approximately 28 hours plus approximately 68.5 hours of IMB-specific incremental components. The substantial difference in incremental contact time reflects the active ingredients of the IMB intervention and is acknowledged as a potential confounder.
